# Rare Missense Functional Variants at *COL4A1* and *COL4A2* in Sporadic Intracerebral Hemorrhage

**DOI:** 10.1212/WNL.0000000000012227

**Published:** 2021-07-20

**Authors:** Jaeyoon Chung, Graham Hamilton, Minsup Kim, Sandro Marini, Bailey Montgomery, Jonathan Henry, Art E. Cho, Devin L. Brown, Bradford B. Worrall, James F. Meschia, Scott L. Silliman, Magdy Selim, David L. Tirschwell, Chelsea S. Kidwell, Brett Kissela, Steven M. Greenberg, Anand Viswanathan, Joshua N. Goldstein, Carl D. Langefeld, Kristiina Rannikmae, Catherine L.M. Sudlow, Neshika Samarasekera, Mark Rodrigues, Rustam Al-Shahi Salman, James G.D. Prendergast, Sarah E. Harris, Ian Deary, Daniel Woo, Jonathan Rosand, Tom Van Agtmael, Christopher D. Anderson

**Affiliations:** From the Center for Genomic Medicine (J.C., S.M., B.M., J.H., J.R., C.D.A.), Department of Neurology (B.M., J.H., S.M.G., A.V., J.R., C.D.A.), McCance Center for Brain Health (J.H., J.R., C.D.A.), and Department of Emergency Medicine (J.N.G.), Massachusetts General Hospital, Boston; Program in Medical and Population Genetics (J.C., J.R., C.D.A.), Broad Institute, Boston, MA; Glasgow Polyomics, Wolfson Wohl Cancer Research Centre, Garscube Campus (G.H.), and Institute of Cardiovascular and Medical Sciences, College of Medical, Veterinary and Life Sciences (G.H., T.V.A.), University of Glasgow, Bearsden, UK; Department of Bioinformatics (M.K., A.E.C.), Korea University, Sejong, South Korea; Stroke Program, Department of Neurology (D.L.B.), University of Michigan, Ann Arbor; Department of Neurology and Public Health Sciences (B.B.W.), University of Virginia Health System, Charlottesville; Department of Neurology (J.F.M.), Mayo Clinic Jacksonville; Department of Neurology (S.L.S.), University of Florida College of Medicine, Jacksonville; Department of Neurology, Stroke Division (M.S.), Beth Israel Deaconess Medical Center, Boston, MA; Department of Neurology, Harborview Medical Center (D.L.T.), University of Washington, Seattle; Department of Neurology (C.S.K.), The University of Arizona, Tucson; Department of Neurology and Rehabilitation Medicine (B.K., D.W.), University of Cincinnati, OH; Center for Public Health Genomics and Department of Biostatistical Sciences (C.D.L.), Wake Forest School of Medicine, Winston-Salem, NC; Centre for Medical Informatics, Usher Institute (K.R., C.L.M.S.), Centre for Clinical Brain Sciences (N.S., M.R., R.A.-S.S.), The Roslin Institute (J.G.D.P.), and Lothian Birth Cohorts Group, Department of Psychology (S.E.H., I.D.), University of Edinburgh; and British Heart Foundation Data Science Centre (K.R.), London, UK. Dr. Anderson is currently at the Department of Neurology, Brigham and Women's Hospital, Boston, MA

## Abstract

**Objective:**

To test the genetic contribution of rare missense variants in *COL4A1* and *COL4A2* in which common variants are genetically associated with sporadic intracerebral hemorrhage (ICH), we performed rare variant analysis in multiple sequencing data for the risk for sporadic ICH.

**Methods:**

We performed sequencing across 559 Kbp at 13q34 including *COL4A1* and *COL4A2* among 2,133 individuals (1,055 ICH cases; 1,078 controls) in United States–based and 1,381 individuals (192 ICH cases; 1,189 controls) from Scotland-based cohorts, followed by sequence annotation, functional impact prediction, genetic association testing, and in silico thermodynamic modeling.

**Results:**

We identified 107 rare nonsynonymous variants in sporadic ICH, of which 2 missense variants, rs138269346 (COL4A1^I110T^) and rs201716258 (COL4A2^H203L^), were predicted to be highly functional and occurred in multiple ICH cases but not in controls from the United States–based cohort. The minor allele of rs201716258 was also present in Scottish patients with ICH, and rs138269346 was observed in 2 ICH-free controls with a history of hypertension and myocardial infarction. Rs138269346 was nominally associated with nonlobar ICH risk (*p* = 0.05), but not with lobar ICH (*p* = 0.08), while associations between rs201716258 and ICH subtypes were nonsignificant (*p* > 0.12). Both variants were considered pathogenic based on minor allele frequency (<0.00035 in European populations), predicted functional impact (deleterious or probably damaging), and in silico modeling studies (substantially altered physical length and thermal stability of collagen).

**Conclusions:**

We identified rare missense variants in *COL4A1*/*A2* in association with sporadic ICH. Our annotation and simulation studies suggest that these variants are highly functional and may represent targets for translational follow-up.

Intracerebral hemorrhage (ICH) accounts for 10%–15% of strokes but is the most fatal and least treatable stroke type.^1-3^ More than half of patients with ICH die within the first year after the disease, and most survivors have prolonged disability.^3^ The molecular pathways underlying ICH remain poorly understood, limiting therapeutic development.

Genome-wide association studies (GWAS) have identified genetic loci associated with ICH risk and outcome.^4,5^ We recently determined a genome-wide significant association for cerebral small vessel disease (CSVD) including nonlobar ICH and small vessel ischemic stroke (SVS) with *COL4A1* and *COL4A2* (collagen IV α chain 1 and 2) at 13q34,^6^ validating previous studies.^7^

Rare mutations that most frequently affect Gly residues in the Gly-Xaa-Yaa repeat in *COL4A1/A2* cause mendelian early-onset cerebrovascular disease, ocular dysgenesis, and myopathy.^8-12^ However, the full spectrum of effects of *COL4A1/A2* mutations remain incompletely characterized.^7,13,14^ Sequencing of a few families or small number of patients (n < 100) suggested rare variants in *COL4A1/A2* can contribute to sporadic ICH.^9-15^

To identify rare coding variants in *COL4A1/A2* that may underlie the aforementioned GWAS association with ICH and SVS, we performed targeted sequencing of the 13q34 region in United States–based studies (1,055 ICH cases and 1,078 ICH-free controls) as well as exonic sequencing of this locus in 192 ICH cases and whole genome sequencing (WGS) of 1,189 ICH-free controls from Scotland. We present annotation results, prediction of deleteriousness, and case/control segregation of rare variants across 13q34 from these datasets.

## Methods

### Standard Protocol Approvals, Registrations, and Participant Consents

The study protocols were approved for enrollment of the United States–based cohorts (Gene Discovery for Warfarin-Related Intracerebral Hemorrhage [GOCHA] and Ethnic/Racial Variation in Intracerebral Hemorrhage [ERICH]) and the Scotland-based cohorts (the Lothian study of Intracerebral Haemorrhage Pathology, Imaging and Neurologic Outcome [LINCHPIN] and Lothian Birth Cohorts [LBC]) by the institutional review boards of Massachusetts General Hospital and University of Edinburgh. Informed consent was obtained from participants or an appropriate legal surrogate according to all recruiting sites.

### Study Participants

#### United States–Based Participants

We collected DNA and phenotyping data on 1,055 patients with ICH (534 lobar and 521 nonlobar ICH) and 1,078 ICH-free controls from GOCHA^5^ and ERICH.^16^ There is no overlap between the participants in the present study and those included in previous targeted sequencing studies of *COL4A1/A2*.^13,17^ A total of 145 participants (18 ICH and 176 controls) in this study appeared in the previous ICH GWAS.^5^

#### Scotland-Based Participants

We collected DNA and phenotyping data on 192 ICH cases (40 lobar and 152 nonlobar) from LINCHPIN among the sample registered in the Edinburgh Lothian Audit of the Treatment of Cerebral Hemorrhage study.^18^

As a control population for the LINCHPIN cohort, we selected clinical and sequencing data from 1,189 individuals from the LBC study as an ethnically and geographically matched stroke-free control population to cases. Self-reported ethnicity of the patients with ICH and controls was white British.^19^ The LBC study recruited people living in Edinburgh and the Lothians who were born either in 1921 or 1936. Individuals who self-reported stroke were removed for this study.

Demographic information for the 2,133 United States–based and 1,381 Scotland-based participants are presented in table 1.

**Table 1:**
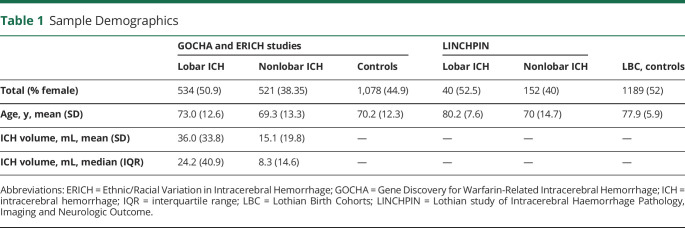
Sample Demographics

### Targeted Sequencing at 13q34 and Quality Control (GOCHA/ERICH and LINCHPIN)

We performed targeted sequencing across 559 Kbp at 13q34 (chr13:110,701,882–111,265,349) including *COL4A1*/*A2* for 2,133 individuals (1,055 ICH cases and 1,078 controls) in GOCHA/ERICH on the 96plex Nimblegen SeqCap platform and for 192 ICH cases in LINCHPIN on the NextSeq 550 platform. The reads with high quality are aligned to the human genome (GRCh37) using BWA (version 0.7.10).^20^ We applied preprocessing steps including duplicate removal, local realignment, indel realignment using Genome Analysis Toolkit (GATK) IndelRealigner, and base quality recalibration using GATK Base Recalibration before the calling. Then GATK best practices pipeline was used for calling the variants from those 2 different platforms, separately. The variants were called and filtered using HaplotypeCaller.

Variants passing the GATK variant quality score recalibration metric were retained. We included only single nucleotide variants or indels with a depth of 10 or higher. We excluded variants with a call rate <0.98, case–control call rate difference >0.005, and differential missingness between cases and controls (*p* < 0.05). Samples were excluded if they had a low average call rate (<0.98), low mean sequence depth (<30), low mean genotype quality (<85), or differential missingness between cases and controls (*p* < 0.05) and Hardy-Weinberg equilibrium test (*p* value < 10^−6^) on cases and controls separately.

### Whole Genome Sequencing (LBC)

WGS of the LBC sample has been performed previously, with sequencing protocol and quality control (QC) metrics as reported.^21^ The variants selected from the GOCHA/ERICH and LINCHPIN studies were further evaluated in the LBC sequencing data as a control population.

### Variant Selection and Annotation

We used the Ensembl variant effect predictor (VEP) software to annotate functional consequences of variants with the Ensembl annotation database on human genome assemble GRCh37.^22^ VEP provides various information of functional consequences of variants (splice acceptor variant, splice donor variant, start lost, stop lost, stop gained, frameshift variant, inframe insertion, inframe deletion, and missense variant) and functional impacts predicted by SIFT (deleterious or tolerate),^23^ PolyPhen (probably damaging, possibly damaging, benign, or unknown),^24^ combined annotation-dependent deletion (CADD),^25^ and LoFtool (0–1).^26^ We also used SnpEff, which categorizes the effects of variants by their genomic locations (e.g., introns, regulatory sites, splicing, missenses, nonsense) into high, moderate, low, or modifier.^27^ We selected nonsynonymous variants with high/moderate by SnpEff, deleterious by SIFT, probably/possibly damaging by PolyPhen, CADD scores >20.0, and LoFtool scores <0.1 in the GOCHA/ERICH and LINCHPIN datasets.

### Single-Variant and Variant-Set Association Tests in GOCHA/ERICH

We used the CATS online calculator^28^ to estimate power to detect ICH association for single variant analysis. We performed single-variant association tests of those variants selected by annotation methods for the ICH risk by subtype (all-mixed, lobar, and nonlobar ICH) using logistic regression in R (r-project.org/). The association models were adjusted for age and sex. Significance thresholds for the single variant test was set as α = 0.05 with Bonferroni correction for the numbers of single variants (number of variants: 39; *p* < 1.28 × 10^−3^).

### Identification and Selection of Potential Causal Rare Variants in ICH (GOCHA/ERICH)

Because our sample size remained extremely underpowered for identifying rare functional variants by conventional association tests, we focused on rare nonsynonymous variants in *COL4A1*/*A2*, particularly those that occur in more than 1 ICH case but not in controls, or in more than 1 control but not in cases. Variants were first explored in GOCHA/ERICH as a discovery dataset and then examined in LINCHPIN and LBC as an external validation of the findings in an independent population.

We applied PLINK^29^ software to assess linkage disequilibrium (LD) and haplotype inferences at this locus, testing whether the common *single nucleotide polymorphism (SNP)*, rs9515201, previously identified in a GWAS of CSVD risk, lies within a haplotype with our identified rare variants.

Because the genetic architectures of lobar and nonlobar ICH are known to differ,^5^ we tested ICH subtype-specific effects for identified variants using the BinomiRare exact test,^30^ which uses Poisson-binomial probabilities to calculate the association strength of variants based on the probabilities of diseased individuals carrying minor alleles of the variants under the null hypothesis that the variants are not associated with the disease. A classical logistic regression analysis was not applied for single-variant test because our selected variants occur only in 1 group (e.g., cases or controls), but not both.^31^ The BinomiRare regression models for ICH subtypes were adjusted for age and sex.

Our identified variants were evaluated using gnomAD^32^ for allele frequencies across populations and Geno2MP (geno2mp.gs.washington.edu/Geno2MP) for phenomic effect in carriers with variants or their family members.^33^

To further assess our selected variants, we also explored the recent whole exome sequencing (WES) of 200,000 individuals in UK Biobank (UKB).^34,35^ We leveraged 137,766 unrelated British White individuals based on kinship relatedness (removing at least 1 of a related pair of individuals) and predicted ancestries from principal components for population structure provided by the UKB.^36^ For ICH-related phenotypes, we used 2 traits in the electric health record including ICD-10 code for ICH (I61; UKB field number 41,202) and “vascular/heart problems diagnosed by doctor” (UKB field number 6,150). The age at onset for these conditions was not available in the UKB database. The differences in the numbers of carriers of our selected variants and noncarriers between cases and controls in the UKB was examined by a χ^2^ test.

### Analysis of Previously Identified Variants on *COL4A1/A2* in GOCHA/ERICH

We investigated coding variants in *COL4A1/A2* previously identified by other sequencing studies in ICH (rs200786329,^13^ rs117412802, rs62621875, and rs201105747^17^) or its related diseases including hereditary angiopathy, nephropathy, aneurysms, and cramps (rs113994104, rs113994105, and rs113994106),^12^ small vessel disease (rs121912857 and rs113994107),^9^ cerebrovascular disease (rs672601346),^37^ and porencephaly (rs113994112,^8^ rs113994114^15^). The information about their positions and amino acid changes are provided in table e-1 (data available from Dryad, doi.org/10.5061/dryad.z34tmpgcq). Previously reported variants that were also identified in our sequencing data were analyzed for association with ICH risk using BinomiRare.

### External Validation of Selected Variants in Scotland-Based Datasets

Selected nonsynonymous rare variants appearing only in cases or controls in the United States–based dataset were explored for validation in LINCHPIN and LBC to determine whether they segregated with cases or controls in these independent datasets. Because LINCHPIN and LBC utilized different recruitment methods and were sequenced using different approaches, discovery of candidate variants in these datasets for validation in the United States–based GOCHA/ERICH cases was not feasible due to concerns for bias.

### In Silico Modeling of Protein Structure and Thermal Stability of Identified Variants

**Figure 1:**
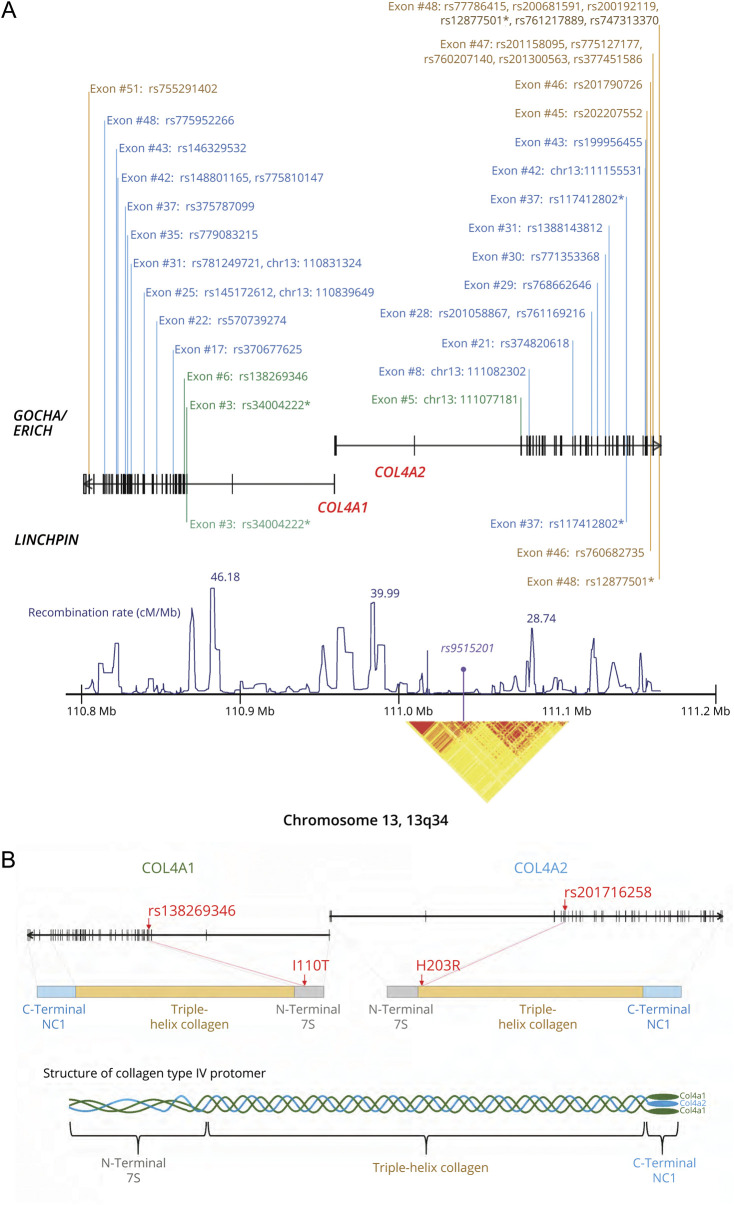
Highly Functional Nonsynonymous Variants Appearing in Patients With Intracerebral Hemorrhage (ICH) Predicted by Various Annotation Methods and Selected *COL4A1* and *COL4A2* Variants (A) Highly functional nonsynonymous variants appearing in ICH cases predicted by annotation methods including SnpEff (high or moderate), SIFT (deleterious), PolyPhen (probably/possibly damaging), combined annotation-dependent deletion (CADD) (>20.0), and LoFtool (>0.1). Green: N-terminal 7S domain; blue: triple helix collagen domain; brown: C-terminal NC domain. *Variants that exist in both datasets. (B) Selected *COL4A1* and *COL4A2* variants. ERICH = Ethnic/Racial Variation in Intracerebral Hemorrhage; GOCHA = Gene Discovery for Warfarin-Related Intracerebral Hemorrhage.

**Figure 2:**
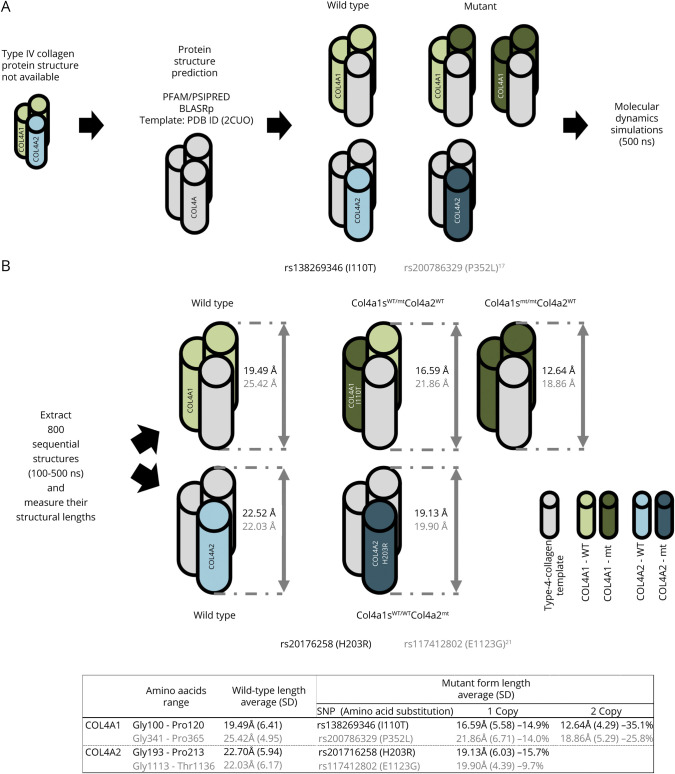
Protein Structure Prediction and Molecular Dynamics Simulation (A) Procedure of protein structure prediction. (B) Comparison of physical lengths of protein prediction models affected by our identified variants including rs138269346 (*CO4A1*) and rs20176258 (*COL4A2*) in black as well as the previously reported variants including rs200786329 (*COL4A1*)^17^ and rs117412802 (*COL4A2*)^21^ in gray. *SNP* = single *nucleotide polymorphism.*

We further assessed structural effects of selected variants on protein structures of *COL4A1/A2* using molecular dynamics (MD) simulation. The α chains in *COL4A1* and *COL4A2* (figure 1A) interact to form 1 triple-helical collagen type IV protomer, α1α1α2(IV), that consists of 3 protein domains: N-terminal 7S, central triple-helical collagen, and C-terminal NC1 (figure 1B). Thus far, the triple-helical collagen structure for α1α1α2(IV) has not been experimentally determined in the Protein Data Bank (PDB; rcsb.org). Therefore, we predicted the tertiary heterotrimer structure of α1α1α2(IV) molecule using protein secondary/tertiary structure prediction tools including PSIPRED,^38^ PFAM,^39^ and BLASTp (blast.ncbi.nlm.nih.gov/Blast.cgi).^40^ Briefly, we used the α1α1α2(IV) structure (PDB ID: 2CUO)^41^ as a template and predicted 2 structure models for our selected variant in *COL4A1* according to the number of copies of its mutations such as COL4A1s^WT/mt^/COL4A2^WT^ and COL4A1s^mt/mt^/COL4A2^WT^ and 1 model for the variant in *COL4A2* such as COL4A1s^WT/WT^/COL4A2^mt^ (figure 2A). To compare structural effects of our variants on α1α1α2(IV), we also generated additional structure models for 2 previously reported variants including rs200786329 (*COL4A1*)^13^ and rs117412802 (*COL4A2*).^17^ These 2 variants were chosen for the comparison because rs200786329^COL4A1^ and rs117412802^COL4A2^ were most recently identified in targeted sequencing of sporadic ICH cases, other than familial early-onset type IV collagen-related diseases. Moreover, our selected variants were located in the same Gly-Xaa-Yaa pattern or the same functional domain. Furthermore, rs200786329^COL4A1^ and rs117412802^COL4A2^ showed strong functional changes in terms of intracellular accumulation and extracellular deficiency of *COL4A1* or *COL4A2* protein levels compared to other variants.^13,17^

MD simulations were performed with the predicted tertiary structures of α1α1α2(IV) molecules containing our identified variants and the 2 previously reported variants using DESMOND (deshawresearch.com/resources_desmond.html)^42^ as described^43^ across 500 nanoseconds (ns). We included a modeling assumption that the atom positions of replaced amino acids stabilized after 100 ns from the start. Maestro (schrodinger.com/maestro; Schrödinger, LLC, 2016) was used to extract and analyze 800 sequential snapshots of simulated protein structures of *COL4A1* and *COL4A2* across 400 ns (100–500 ns).

In addition, the effect of the tripeptide composition (Gly-Xaa-Yaa) containing our identified variants on collagen triple helix stability was estimated using the collagen thermal stability calculator (compbio.cs.princeton.edu/csc),^44^ which predicts the melting temperature (*T*_*m*_).

### Data Availability

Sequencing data used in this study are available on dbGAP (ncbi.nlm.nih.gov/gap/; accession ID: phs000416.v2.p1). Additional data supporting these findings are available by the authors upon reasonable request.

## Results

### Identification and Genetic Association Tests of Rare Variants in *COL4A1/A2*

Following targeted sequencing and stringent QC of the 13q34 region in the United States–based GOCHA/ERICH studies, we retained 1,055 patients with ICH and 1,078 ICH-free controls with 11,815 variants. Among these variants, 0.91% were nonsynonymous (106 missense, 1 nonsense) and 0.70% were synonymous coding variants.

In the Scotland-based LINCHPIN study, 192 patients with ICH remained after QC with 1,380 variants at the 13q34 locus. From these, 1.01% were nonsynonymous (15 missense and 1 nonsense) and 1.88% were synonymous coding variants.

Selecting for nonsynonymous variants with high/moderate impact by SnpEff, deleterious predicted by SIFT, probably/possibly damaging predicted by PolyPhen, CADD scores >20.0, and LoFtool scores >0.1, we were left with 39 variants in GOCHA/ERICH and 4 variants in LINCHPIN, with 3 of these missense variants, including rs34004222, rs117412802, and rs12877501, common between studies. These nonsynonymous variants, predicted to be highly functional based on multiple annotation methods, are shown in figure 1A. None of these variants is in significant LD (*r*^*2*^ > 0.4) with rs9515201, the leading common variant that we previously identified in a recent CSVD GWAS.^6^ Detailed information on these variants appears in table e-2 (data available from Dryad, doi.org/10.5061/dryad.z34tmpgcq). Annotation information for all detected variants in both sequencing datasets is described in detail in table e-3 (GOCHA/ERICH) and table e-4 (LINCHPIN) (data available from Dryad, doi.org/10.5061/dryad.z34tmpgcq).

According to our statistical power calculation (figure e-1, doi.org/10.5061/dryad.z34tmpgcq), we have extremely limited power (less than 20%) for detecting genetic association of such a rare variant (minor allele frequency [MAF] <0.001) with risk of sporadic ICH. We therefore limited single variant tests to those 39 variants but did not observe a variant with significant (*p* < 1.2 × 10^−3^) association with ICH subtypes (table e-5, data available from Dryad, doi.org/10.5061/dryad.z34tmpgcq).

### Selection of Potential Causal Variants in ICH (GOCHA/ERICH)

Due to this expected low statistical power for single rare variant discovery in the United States–based GOCHA/ERICH dataset, we chose to focus on nonsynonymous mutations that appear in at least 2 participants exclusively in 1 group (e.g., ICH) but is absent in the other group (e.g., ICH-free controls). We did not observe any nonsynonymous variants that occurred in at least 2 controls but not in cases, but we did identify 2 rare missense variants (rs138269346 and rs201716258) appearing in at least 2 ICH cases without occurrence in the controls (table 2). rs138269346 (*COL4A1* Ile110Thr) is in exon 5 of *COL4A1* corresponding to the N-terminal 7S domain and rs201716258 (*COL4A2* His203Leu) is in exon 10 of *COL4A2* corresponding to the triple-helix collagen domain where it affects an X residue of the Gly-Xaa-Yaa repeat (figure 1B). rs138269346^COL4A1^ was present in 4 ICH cases (2 lobar and 2 nonlobar ICH cases) and rs201716258^COL4A2^ was observed in another 2 ICH cases (1 lobar and 1 nonlobar ICH case). These ICH cases were each heterozygous for the minor alleles of these variants and no individual carried the minor alleles of both variants. These variants were not associated with age at disease onset, sex, or ICH volume (table e-6, data available from Dryad, doi.org/10.5061/dryad.z34tmpgcq).

**Table 2:**
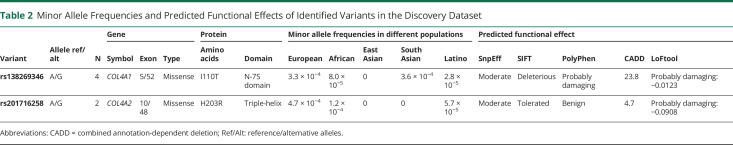
Minor Allele Frequencies and Predicted Functional Effects of Identified Variants in the Discovery Dataset

According to our LD calculation and haplotype analysis, these 2 rare variants are not in LD (*r*^*2*^ < 0.01) with the lead SNP of our previous GWAS, rs9515201,^6^ and we did not observe haplotypes carrying minor alleles of the identified rare variants and rs9515201 (table e-7, data available from Dryad, doi.org/10.5061/dryad.z34tmpgcq).

### Functional Annotations of rs138269346 and rs201716258

Based on gnomAD, these 2 variants are rare in European populations (MAF <0.0005) and even rarer in other populations (table 2). Rs138269346^COL4A1^ was deemed pathogenic due to its annotation across multiple prediction tools: moderate by SnpEff, deleterious by SIFT, probably damaging by PolyPhen, and probably damaging by LoFtool. It showed a very high CADD score of 23.8, suggesting that this variant is among the top 0.42% (10^−2.38^) of most predicted deleterious variants in the human genome. The other variant, rs201716258^COL4A2^, was also considered to have a negative effect by SnpEff (moderate) and LoFtool (probably damaging), but not by SIFT (tolerated), PolyPhen (benign), and CADD (4.7).

### Validation of rs138269346 and rs201716258 in Scotland-Based Participants

We set out to validate our identified variants using the independent LINCHPIN dataset. We detected 2 patients with ICH who carry the minor allele of rs201716258^COL4A2^ but no carriers for rs138269346^COL4A1^. For rs201716258, both individuals developed nonlobar ICH in the right ventricle (ICH epicenter was lentiform) with a low small vessel disease score.^45^ The ages of patients (1 male, 1 female) were 85 and 73 at the time of ICH, and there was no history of ischemic stroke.

Turning to the population-based LBC cohort, we identified 2 ICH-free controls who carry the minor allele of rs138269346^COL4A1^ but no carriers for rs201716258^COL4A2^. One of these 2 ICH-free controls carrying the rs138269346^COL4A1^ in LBC self-reported a history of hypertension and myocardial infarction.

### Further Evaluation of rs138269346 and rs201716258 in Geno2MP and UKB

Within individuals contributing to the Geno2MP database, carriers of these variants expressed phenotypic abnormalities previously identified in patients with rare collagen IV mutation syndromes.^14^ In Geno2MP, we found 5 heterozygous rs138269346^COL4A1^ carriers and 6 heterozygous rs201716258^COL4A2^ carriers (table e-8, data available from Dryad, doi.org/10.5061/dryad.z34tmpgcq). One of the rs138269346^COL4A1^ carriers had multiple abnormalities in the eyes and the cardiovascular and nervous systems, and relatives of the other 4 carriers also had abnormalities in the eye, musculature, and cardiovascular and nervous systems. Two of rs201716258^COL4A2^ carriers had abnormalities in the ears and the cardiovascular system, respectively, and the relatives of the other 4 carriers also had abnormalities in the nervous system or musculature.

Among the 137,766 unrelated individuals in the 200K WES (table e-9), we found 1 rs138269346 carrier but none for rs201716258 out of 276 proxy patients with ICH (by ICD-10 code I61). Out of 41,032 individuals with vascular/heart problems, there are 33 carriers for rs138269346 and 63 carriers for rs201716258. According to our χ^2^ analysis, rs138269346 was nominally associated with the proxy ICH (χ^2^ = 3.4; *p* = 0.063) although there is only 1 proxy ICH case carrying rs138269346. We did not observe significant association in the rest of the χ^2^ tests (table e-10, data available from Dryad, doi.org/10.5061/dryad.z34tmpgcq).

### Genetic Association Tests With ICH Subtypes

To determine which ICH subtype is relevant to these variants, we employed BinomiRare (table 3), which revealed that rs138269346^COL4A1^ is nominally associated with risk for nonlobar ICH (*p* = 0.054) and less associated with lobar ICH (*p* = 0.077) and all-mixed ICH (*p* = 0.090). However, rs201716258^COL4A2^ did not carry a significant association with any ICH subtype (all *p* values > 0.12).

**Table 3:**
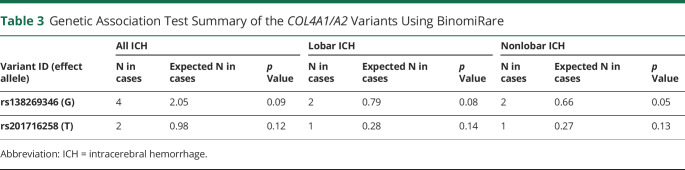
Genetic Association Test Summary of the *COL4A1/A2* Variants Using BinomiRare

Among the previously reported variants in *COL4A1/A2*, we could detect 3 nonsynonymous variants in *COL4A2*, including rs117412802, rs62621875, and rs201105747, which were previously reported in only patients with ICH and not in controls.^17^ However, in the GOCHA/ERICH dataset, we found carriers of these variants in both ICH cases (carriers of rs117412802: 20; and rs62621875: 1) and controls (rs117412802: 24; and rs62621875: 1). We identified rs201105747 only in 1 control. From the LINCHPIN dataset, we found only rs117412802 among 4 patients with ICH (3 lobar and 1 nonlobar ICH). We found no significant associations of these observed variants with ICH risk from BinomiRare (table e-11, data available from Dryad, doi.org/10.5061/dryad.z34tmpgcq).

### In Silico Functional Analysis of rs138269346 and rs201716258

To gain insight into any structural potential consequences of these 2 missense variants, we performed MD simulations. Based on α chain composition of α1α1α2(IV), each protomer can contain 1 or 2 variant α1(IV) molecules and 1 α2(IV). We therefore constructed 2 structural models for rs138269346^COL4A1^: COL4A1s^WT/I110T^/COL4A2^WT^ and COL4A1s^I110T/I110T^/COL4A2^WT^. Next, for rs201716258^COL4A2^, 1 structure model was predicted: COL4A1s^WT/WT^/COL4A2^H203L^ (figure 2A). From these simulations, we observed that 2 mutation structures of rs138269346^COL4A1^, COL4A1s^WT/I110T^/COL4A1^WT^ and COL4A1s^I110T/I110T^/COL4A2^WT^, result in average lengths of 16.59 ångströms (Å; equal to 10^−10^m) (SD 5.58 Å) and 12.64 Å (SD 4.29 Å), respectively (figure 2B). These are 14.9% and 35.1% shorter than the average length of the wild type (average 19.49 Å; SD 6.41 Å). Likewise, we also found that the mutated structure for rs201716258^COL4A2^, COL4A1^WT/WT^/COL4A2^H203L^ (average 19.13 Å; SD 6.03 Å), became 15.7% shorter than the wild type (average 22.70 Å; SD 5.94 Å).

For comparison, we also generated structural models of the previously reported *COL4A1/A2* variants rs200786329^COL4A1^ and rs117412802^COL4A2^. Interestingly, we found the same pattern in the simulations of the structure models with mutations of the previously reported variants. The average lengths of those mutant forms for rs200786329^COL4A1^ and rs117412802^COL4A2^ are 9.7%–25.8% shorter than their wild types (figure 2B).

It is well established that the amino acid sequence of the collagenous domain and mutations in collagen influence the thermal stability and melting temperature of the triple helix.^46^ According to the collagen stability calculator,^44^ COL4A1^I100T^ reduces predicted *T*_*m*_ by 2.2°C compared to COL4A1^wt^, while COL4A2^H203L^ increases the *T*_*m*_ by 2.1–3.1°C compared to COL4A2^wt^ (figure e-2, data available from Dryad, doi.org/10.5061/dryad.z34tmpgcq), further lending support to their functionality.

## Discussion

We conducted a targeted sequencing study of 1,055 ICH cases and 1,078 controls, and an additional dataset (192 ICH cases) at the *13q34* locus, which we and others have previously identified in genetic association studies for ICH and related manifestations of CSVD.^6,7^ 13q34 has been the target of prior sequencing studies in ICH, but the sample size in this current study (n = 2,325) is approximately 10 times larger than those prior efforts (n < 200).^8,9,12,13,15,17,37^ However, even with this boost in sample size, statistical power remains low for rare variants, so we focused on nonsynonymous variants appearing only in ICH cases but not in controls to restrict our search to the most likely pathogenic variants. Our study identified 2 novel rare missense variants in *COL4A1*/*COL4A2*, appearing in cases only in our discovery dataset that (1) are predicted to have damaging effects, (2) are not in LD with common SNP identified in the previous GWAS for CSVD, and (3) are predicted to substantially alter the physical length and the thermal stability of the type IV collagens. Furthermore, despite the limited statistical power of our sample size, we did observe a nominally significant association at rs138269346^COL4A1^ for nonlobar ICH risk.

In our attempt to externally validate these results in independent datasets, we detected 2 patients with ICH carrying rs201716258^COL4A2^ in LINCHPIN. No ICH case carriers for rs138269346^COL4A1^ were found, although 2 individuals with this variant were identified in the LBC control population. Given the size difference between the case populations in the 2 studies and the differences in recruitment between the case/control datasets and LBC, the significance of this replication failure at rs138269346^COL4A1^ is unclear.

The majority of mendelian disease–associated mutations in *COL4A1/A2* identified to date affect the Gly residue in the Gly-Xaa-Yaa repeat pattern, which is necessary for stable triple-helix formation, and more mutations have been described in *COL4A1* than *COL4A2*.^14^ However, the amino acids of our selected variants are located on the X residue in the pattern. Among the previously identified mutations for sporadic ICH, P352L^rs200786329^ (*COL4A1*)^13^ and E11223G^rs117412802^ (*COL4A2*)^17^ are also located on non-Gly residue in the tripeptide pattern, the Y and X residues, respectively. These previous variants (rs200786329 and rs117412802) significantly reduced the ratio of extracellular to intracellular *COL4A1* or *COL4A2* proteins compared to wild types in cultured cells, which is considered as a potential pathogenic mechanism underlying the type IV collagens related diseases.^13,17^ Interestingly, our MD simulation shows that these previous variants (rs200786329^13^ and rs117412802^17^) as well as our new variants (rs138269346 and rs20176258) substantially shorten the physical lengths of triple-helical structures, indicating that altered protein structures such as shortening of the helical domains could lead to changes in the protein functions including secretion of type IV collagen into extracellular matrix or secretion of mutant protein. In this regard, our thermal stability study predicted that COLl4A1^I100T^ decreases (−2.2°C) and COL4A2^H203L^ increases (+2.1–3.1°C) *T*_*m*_ of collagen structures. These changes are not small in magnitude compared to the 2°C reduction affected by mendelian disease–associated Col2a1 mutations.^47^ Therefore, it also may be possible that our identified variants exert their effects on type IV collagen function through affecting the thermal stability of collagen, analogous to other collagen mutations.

Our study has several limitations. First, due to limited statistical power, we focused on rare variants appearing only in ICH cases but not controls, which could be considered conservative and increase false-negative associations for variants with low penetrance. Furthermore, we lack an analogous case/control ICH dataset with deep sequencing for direct validation of our observed variants. Targeted sequencing of 13q34 in LINCHPIN allowed us to verify the existence of rs201716258 in ICH cases, but the relatively small sample size and substantial difference in proportions of ICH subtypes compared to the discovery dataset makes the lack of observation of rs138269346 inconclusive. The population-based subjects from LBC are a welcome adjunct to the LINCHPIN ICH cases, but the varying burden of ICH-relevant disease histories and comorbidities and prospective nature of the LBC study, which is not continually updated, make interpretation of the appearance of rs138269346 in ICH-free individuals challenging. Due to the lack of genome-wide genotyping on the vast majority of subjects, population structure could not be assessed and adjusted for in our single variant association tests. While interesting and supportive of known phenotypic associations in rare *COL4A1/A2* syndromes, care must be taken in interpretation of phenotypes of variant carriers in Geno2MAP as this database is not a representative population sample and does not provide data to permit testing of statistical enrichment of variants. We observed differences in predicted effects of our identified variants across annotation methods. However, annotation methods could generate substantially different predictions of variant effects due to variation in the weighting of variant conservation and other features.^48,49^ Because of their rarity, imputation of these variants in existing ICH GWAS datasets using the HRC and TOPMed reference panels return very poor imputation quality scores, preventing additional forms of replication at this time.

Furthermore, there are challenges in integrating UKB data into our study. First, heterogeneous phenotyping of ICH cases in the UKB is a major concern that could substantially affect our genetic models.^50^ For example, ICH status based on the ICD-10 code in electric health records is less precise than manual phenotyping of ICH cases due to a lack of distinction between sporadic primary ICH and secondary ICH caused by trauma, brain tumor, hemorrhagic transformation of ischemic stroke, vascular malformation, and other factors, even among health care practitioners. In addition, the relatively young age, low number of ICH cases, and lack of ICH adjudication in the UKB make it ill-suited as a validation dataset for this application.

This work marks the continued progression of genetic research into collagen IV from gene mapping in mendelian disease through to identification of risk variants for sporadic disease in the general population. These sequencing results build on extant GWAS of ICH, demonstrating that *COL4A1* and *COL4A2* contribute to sporadic ICH not only through as-yet poorly understood mechanisms related to associations at common variants but also potentially by rare variants that alter protein structure. Our observations from both sequenced datasets as shared through this article add substantially to our understanding of the burden and spectrum of *COL4A1*/*COL4A2* rare variation in sporadic ICH. Given the significance of 13q34 in both rare and common variant studies of ICH, studies building on our observations may link the pathogenic processes that underlie monogenic and polygenic ICH risk at this locus.

## Glossary

CADD: combined annotation-dependent deletion
CSVD: cerebral small vessel disease
ERICH: Ethnic/Racial Variation in Intracerebral Hemorrhage
GATK: Genome Analysis Toolkit
GOCHA: Gene Discovery for Warfarin-Related Intracerebral Hemorrhage
GWAS: genome-wide association studies
ICD-10: International Classification of Diseases–10
ICH: intracerebral hemorrhage
LBC: Lothian Birth Cohorts
LD: linkage disequilibrium
LINCHPIN: Lothian study of Intracerebral Haemorrhage Pathology, Imaging and Neurologic Outcome
MAF: minor allele frequency
MD: molecular dynamics
ns: nanoseconds
PDB: Protein Data Bank
QC: quality control
SNP: single nucleotide polymorphism
SVS: small vessel ischemic stroke
UKB: UK Biobank
VEP: variant effect predictor
WES: whole exome sequencing
WGS: whole genome sequencing
